# Evaluation and Comparison of the Processing Methods of Airborne Gravimetry Concerning the Errors Effects on Downward Continuation Results: Case Studies in Louisiana (USA) and the Tibetan Plateau (China)

**DOI:** 10.3390/s17061205

**Published:** 2017-05-25

**Authors:** Qilong Zhao, Gabriel Strykowski, Jiancheng Li, Xiong Pan, Xinyu Xu

**Affiliations:** 1 School of Geodesy and Geomatics, Wuhan University, Wuhan 430079, China; jcli@sgg.whu.edu.cn (J.L); xyxu@sgg.whu.edu.cn (X.X.); 2 National Space Institute, Technical University of Denmark, Copenhagen Ø 2800, Denmark; gs@space.dtu.dk; 3 Key Laboratory of Geospace Environment and Geodesy of Ministry of Education, Wuhan University, Wuhan 430079, China; 4 Faculty of Information Engineering, China University of Geosciences, Wuhan 430074, China; pxjlh@cug.edu.cn

**Keywords:** systematic error, random errors, downward continuation, Louisiana, Tibetan Plateau, semi-parametric, regularization

## Abstract

Gravity data gaps in mountainous areas are nowadays often filled in with the data from airborne gravity surveys. Because of the errors caused by the airborne gravimeter sensors, and because of rough flight conditions, such errors cannot be completely eliminated. The precision of the gravity disturbances generated by the airborne gravimetry is around 3–5 mgal. A major obstacle in using airborne gravimetry are the errors caused by the downward continuation. In order to improve the results the external high-accuracy gravity information e.g., from the surface data can be used for high frequency correction, while satellite information can be applying for low frequency correction. Surface data may be used to reduce the systematic errors, while regularization methods can reduce the random errors in downward continuation. Airborne gravity surveys are sometimes conducted in mountainous areas and the most extreme area of the world for this type of survey is the Tibetan Plateau. Since there are no high-accuracy surface gravity data available for this area, the above error minimization method involving the external gravity data cannot be used. We propose a semi-parametric downward continuation method in combination with regularization to suppress the systematic error effect and the random error effect in the Tibetan Plateau; i.e., without the use of the external high-accuracy gravity data. We use a Louisiana airborne gravity dataset from the USA National Oceanic and Atmospheric Administration (NOAA) to demonstrate that the new method works effectively. Furthermore, and for the Tibetan Plateau we show that the numerical experiment is also successfully conducted using the synthetic Earth Gravitational Model 2008 (EGM08)-derived gravity data contaminated with the synthetic errors. The estimated systematic errors generated by the method are close to the simulated values. In addition, we study the relationship between the downward continuation altitudes and the error effect. The analysis results show that the proposed semi-parametric method combined with regularization is efficient to address such modelling problems.

## 1. Introduction

Airborne gravimetry is a cost efficient way of collecting gravity data in mountainous and uninhabited areas [1,2,3,4,5,6]. The downward continuation of airborne gravity data from the flight altitude to the surface is frequently used in the geophysical/geodetic applications, i.e., contemporary geoid models [3,4,5,6,7,8,9,10,11,12,13,14,15,16,17,18,19,20]. The mathematical formulation of the upward/downward continuation of potential fields is based on the Abel-Poisson’s integral generated from a Fredholm integral equation of the first kind. The downward continuation that inverts the solution of Abel-Poisson’s integral, has become a standard strategy applied by many authors. Jacobi’s iterations are proposed to compute decomposition of the inverse Poisson’s integral [14]. The problem was also treated using Tikhonov’s regularization [21]. Other downward continuation methods include least-squares collocation [21]. 

The downward continuation is an error amplification process. Therefore downward continued gravity signals using the inverse Poisson’s integral are easily destroyed by the observation errors. Airborne gravimetry data are affected by all kinds of errors. For instance, air turbulence generates random errors, and data from gravity sensors contain systematic errors [2,22]. The above factors vary with the change of regions, sensors and the weather conditions. The air turbulence generates high frequency systematic and random errors, which can be reduced by the low-pass filtering [2,3,5,6,8,23]. In the actual survey conditions, the errors in the airborne gravity data can be classified as random and systematic errors. The systematic errors are often removed using the crossover adjustment [2,3,6,7,8,11]. However, it is not a workable solution in the USA National Geodetic Survey (NGS) Alabama and Louisiana projects due to an apparent systematic error of the cross lines [14,24] (ftp://ftp.ngs.noaa.gov/pub/grav-d/CS02/). Sometimes airborne data are collected to offset limitations of the existing terrestrial gravity database. Therefore we cannot use the terrestrial gravity data for removing the errors in the airborne data in many mountainous areas like the Tibetan Plateau [7]. We find the effective noise-removal techniques using the global model for that area. In addition, the flight altitudes of the airborne gravimetry and, thus, the altitude from which the data are downward continued, can be very different. For example, the extreme altitude in the USA Gravity for the Redefinition of the American Vertical Datum program (GRAV-D) is approximately 11 km. That is also the planned flight altitude for the future Tibetan Plateau airborne gravimetry project to avoid the aviation hazards. Therefore the relationships between the downward continuation from the flight altitude and the error effects of random and systematic errors should be analyzed for the Tibetan Plateau.

The problem of the amplification of the random errors by the downward continuation can be minimized by regularization methods in the space-domain or by the filtering in the frequency-domain [3,10,11,21]. However, the systematic errors are difficult to remove when there are no external surface gravity data as is the case for the Tibetan Plateau. We propose to use the semi-parametric model to estimate and to remove the systematic errors. The semi-parametric method includes a parametric and a non-parametric component [25,26,27,28,29,30,31,32,33,34,35]. It is a statistical method for systematic errors, which has been applied in the fields of electrical engineering [36]. However, the “penalized likehood” allows one to estimate the systematic components without any a priori knowledge. Fessler presented the rationale behind “the penalized likehood” estimation in [26,27]. To the authors’ knowledge, there are no papers dealing with airborne gravimetry that use the semi-parametric model for the estimation of the systematic errors. The relation between the method proposed in this paper (the semi-parametric method) and the least-squares collocation as well as a serious study of the pros and cons of both methods is outside the scope of the present paper. It is, nevertheless, an obvious topic for future studies. The use of the least-squares–collocation for downward continuation of aerogravity data requires, to the authors’ knowledge, a full processing at flight height prior to downward continuation. The semi-parametric method offers a possibility of direct downward continuation from the flight height to the surface from the measured data and without the a-priori knowledge of the error characteristics.

The objective of this paper is to analyze the effect of systematic and random errors on downward continuation. We focus on the following issues: (1) the effectiveness of the semi-parametric model combined with the regularization in removing the errors on the Louisiana-project data; (2) the effectiveness of the semi-parametric method in modeling the systematic errors in the Tibetan plateau; (3) the relationship between the errors and the flight altitude in downward continuation (11 km for the Louisiana project as well as for the future Tibetan Plateau project). The observed airborne gravity data in the NGS Louisiana project and the simulated gravity disturbances from the EGM08 for the Tibetan Plateau will be used to assess the performance of the above methods on the error reduction.

## 2. The Semi-Parametric Method Combined with the Regularization

The proposed error handling method is based on the inverse Poisson’s integral where the systematic errors are removed using the semi-parametric method while the random errors are reduced using the regularization method.

### 2.1. The Inverse Poisson’s Integral

Downward continuation can be viewed as the inverse operation of the Poisson’s integral, which itself is a solution to the first boundary value problem of potential theory. In the spherical approximation, the upward continuation Poisson’s integral yields:

(1) $$\delta g^{air}(r,\varphi ,\lambda )=\frac{R^{2}(r^{2}-R^{2})}{4\pi r}\int_{\varphi \prime =-\frac{\pi }{2}}^{\frac{\pi }{2}}\int_{\lambda \prime =0}^{2\pi }\frac{\delta g^{g}(R,\varphi \prime ,\lambda \prime )}{l^{3}(r,\varphi ,\lambda ;R,\varphi \prime ,\lambda \prime )}\cos\varphi \prime d\varphi \prime d\lambda \prime$$

where $\delta g^{air}(r,\varphi ,\lambda )$ is the gravity disturbance at a field point with the spherical latitude φ, the longitude λ, r=R+h, and *h* is the flight altitude. *R* is the mean radius of the Earth. $\delta g^{g}(R,\varphi \prime ,\lambda \prime )$ is the gravity disturbance at the variable integration point on the geoid. l(r,φ,λ;R,φ′,λ′) is the distance between the fixed field point and the variable integration point. Gravity disturbance can be generated by $\delta g\;=\;g_{P}-\;\gamma_{P}$. $g_{P}$ is the gravity value. The normal gravity $\gamma_{P}$ is calculated by $\gamma_{P}\;=\;978031.85\;\times \;(1+0.005278895\;\times \;\sin{\left(\phi \right)}^{2}\;+\;0.000023462\;\times \;\sin{\left(\phi \right)}^{4})$ in NGS GRAV-D general airborne gravity data use manual [14] (ϕ is the geodetic latitude).

The distance between the field point and the source point l is calculated as follows:

(2) $$l=\sqrt{r^{2}+R^{2}-2rR\cos\psi }$$

where ψ is the spherical distance between the two points on the surface of a unit sphere, represented by (φ, λ) and (φ′, λ′), so that:

(3) cosψ = sinφsinφ′ + cosφcosφ′cos(λ−λ′)

Equation (1) is called the Poisson’s integral formula. Normally the discrete Poisson’s integration is used for the downward continuation. We use the matrix-vector notation for a grid representation of the Poisson’s integral model which can be written as follows [10,11,12,21,36]:

(4) $$\delta g^{air}=B\delta g^{g}$$

where $\delta g^{air}$ is a vector of dimension M of the gravity disturbances in the air. $\delta g^{g}$ is a vector of length N of the gravity disturbances, containing point disturbances $\delta g^{g}$ on the ellipsoid, and the M×N matrix B consists of elements $b_{ij}$ explained below.

The diagonal entries of are given by:

(5) $$b_{ii}=\frac{R}{4\pi r_{i}}\{2\pi [\frac{r_{i}+R}{r_{i}}(1-\frac{r_{i}-R}{l(r_{i},\psi_{0},R)})]-\;\sum_{j=1}^{i-1}R\frac{\left(r^{2}-R^{2}\right)}{l^{3}(r,\psi ,R)}\Delta \sigma_{j}-\;\sum_{j=i-1}^{N_{c}}R\frac{\left(r^{2}-R^{2}\right)}{l^{3}(r,\psi ,R)}\Delta \sigma_{j}\}$$

where $\Delta \sigma_{j}$ is the surface element centered at the jsth geographical node $\sigma_{j}$. $N_{C}$ is the number of data within the spherical cap of radius 1°, $\psi_{0}$ is the radius of the inner zone.

The off-diagonal elements of are given as follows:

(6) $$b_{ij}=\{\begin{array}{cc} \frac{R^{2}}{4\pi r_{i}}\frac{\left(r^{2}-R^{2}\right)}{l^{3}(r,\psi ,R)}\Delta \sigma_{j} & \psi_{ij}\leq \psi_{0}\\ 0 & \psi_{ij}>\psi_{0} \end{array}$$

### 2.2. The Semi-Parametric Method

The parametric model only have parameters but the semi-parametric model has two types: the parameters are modeled using the empirical or mathematical equations; the nonparametric parts whose relationship with the observations is unknown, are handled by the nonparametric method.

To describe the nonparametric method, initially suppose that the data consist of the n coupled pairs $\left(t_{i},y_{i}\right)$, i=1,⋯,n, related by the simple model:

(7) $$y_{i}=f\left(t_{i}\right)+e_{i}$$

where f is a function on the interval (a,b) and the residuals $e_{i}$ have the properties:

(8) $$E\left(e_{i}\right)=0,\mathrm{var}\left(e_{i}\right)=E\left(e_{i}^{2}\right)=\sigma^{2},E\left(e_{i}e_{j}\right)=0\;i\neq j,\;\mathrm{all}\;i$$

E is the mathematical expectation. Rather than considering some arbitrarily chosen parametric form, for example $f\left(t\right)=a+bt+ct^{2}$, the problem considered here is to estimate f(t) by a nonparametric method.

The nonparametric relation becomes:

(9) $$L_{i}=b_{i}^{T}X+s(t_{i})+\Delta_{i}\;i=1,\cdots ,n$$

where $L_{i}$ is the observation including the errors. X is the estimated value. $s(t_{i})$ is a systematic error which is the nonparametric function. $\Delta_{i}$ is a random error vector. We consider as s(t), and as an approximation to the original systematic error function, the cubic smoothing spline, which for a given $\alpha_{S}\geq 0$, minimizes:

(10) $$\frac{1}{n}\sum_{i=1}^{n}P_{i}{\left(L_{i}-b_{i}^{T}X-s(t_{i})\right)}^{2}+\alpha_{S}\int_{t_{1}}^{t_{n}}{\left(s\prime \prime (t)\right)}^{2}dt=\min$$

s″(t) is the second order derivative of s(t). We call this the semi-parametric regression model or “penalized likehood” estimation. The first term of Equation (10) penalizes the lack of goodness of fit of the function to the data, and the second penalizes the lack of smoothness of the approximating function. The solution to Equation (10) is unique at every data point $t_{i}$. More importantly, the semi-parametric model can estimate the systematic error without the external data, because the “penalized likehood” estimation allows the data “to speak for itself” without the a priori knowledge [26,27,28]. The key point is to use $\alpha_{S}$ to balance the two terms relative to each other. By varying $\alpha_{S}$, the smoothness of s(t) is varied. At the extremes, when $\alpha_{S}$ goes to infinity, the left hand side of Equation (10) is forced to be linear over the whole range of t values and is then the best least squares line through the data. When $\alpha_{S}\to 0$, y tends to be an interpolating function for the data, fitting every data point exactly. The generalized cross validation method to calculate $\alpha_{S}$ is used.

Smoothing splines were originally proposed by Whittaker [34], Schoenberg [32], and Reinsch [29]. The analysis of their statistical properties, when s(t) are periodic, appears in Wahba [33] and Rice and Rosenblatt [30,31]. An analysis of the nonperiodic case appears in Rice and Rosenblatt [31]. The character of the function s minimizing this expression is not clear, although in the case of the direct observation of X, $b_{i}^{T}X=f\left(t_{i}\right)$, the solution can be shown to be a natural cubic spline. For the natural cubic splines, the specific derivation is given in [10,12,21,28]. The most important relations are summarized below:

(11) $$\int_{t_{1}}^{t_{n}}{\left(s\prime \prime (t)\right)}^{2}dt={\hat{s}}^{T}FG^{-1}F^{T}s$$

where F and G are n×(n−2) and (n−2)×(n−2) band matrices, respectively.

(12) $$F_{ij}=\{\begin{array}{cc} h_{j}^{-1}, & i=j\\ -(h_{j}^{-1}+h_{j+1}^{-1}), & i=j+1\\ h_{j+1}^{-1}, & i=j+2\\ 0 & others \end{array}$$

(13) $$G_{ij}=\{\begin{array}{cc} 1/3(h_{i-1}+h_{i}) & i=j,j=2\cdots n-1\\ 1/6h_{i+1} & i=j-1,j=2\cdots n-2\\ 1/6h_{i} & i=j+1,j=1\cdots n-3\\ 0 & others \end{array}$$

i=1,2,⋯,n,j=1,2,⋯,n−2

where $h_{i}=t_{i+1}-t_{i}$ (i=1,2,⋯,n−1). According to the Lagrange extreme value, the functions are constructed as follows:

(14) $$\phi =V^{T}PV+\alpha_{S}{\hat{s}}^{T}R_{s}\hat{s}+2{K_{r}}^{T}(B\hat{X}+\hat{s}-L-V)$$

where $R_{s}=FG^{-1}F^{T}$, *P* is the weight matrix of observations. $K_{r}$ is the Lagrange constant. Adjustment calculation is omitted. The final results are as follows:

(15) $$\hat{X}={\left(B^{T}P(I-M)B\right)}^{-1}(B^{T}P(I-M)L)$$

(16) $$\hat{s}={\left(P+\alpha_{S}R_{s}\right)}^{-1}(PL-B^{T}P\hat{X})$$

where $M={\left(P+\alpha_{S}R_{s}\right)}^{-1}P$. The generalized cross validation method of the semi-parametric model was used to calculate the smoothing parameter $\alpha_{S}$. It is defined as:

(17) $$GCV(\alpha_{S})=\frac{V^{T}PV}{{\left(1-tr(H(\alpha_{S}))/n\right)}^{2}}$$

where $H={\left(B^{T}P(I-M)B\right)}^{-1}B^{T}P(I-M)$.

### 2.3. The Regularization Method

The Tikhonov regularization is a classic regularization method. Minimizing the Tikhonov cost function for the downward continuation yields [10,12,21]:

(18) $$\delta g_{dwc}={\left(B^{T}PB+\alpha_{R}I\right)}^{-1}B^{T}PL$$

where $\alpha_{R}$ is the regularization parameter and I is the unit matrix. The regularization parameter in this study is determined by the generalized cross-validation:

(19) $$\alpha_{R}=\arg\min\frac{m{\left\|B\hat{X}-L\right\|}^{2}}{{\left(trace(I-Q_{\alpha_{R}})\right)}^{2}}$$

where argmin means the argument of the minimum. *m* is the number of measurements and $Q_{\alpha_{R}}$ is

(20) $$Q_{\alpha_{R}}=\;B{\left(B^{T}B+{\alpha_{R}}^{2}I\right)}^{-1}B^{T}$$

### 2.4. The Semi-Parametric Method Combined with Regularization

For the purpose of dealing with the systematic errors and the random errors we combine the two methods for the downward continuation: The semi-parametric model is used to estimate the systematic errors; then we apply the regularization method to suppress the random errors.

The final equation is as follows:

(21) $$\delta g^{g}(R,\varphi \prime ,\lambda \prime )={\left(B^{T}PB+\alpha_{R}I\right)}^{-1}B^{T}P(\delta g^{air}(r,\varphi ,\lambda )-\hat{s})$$

where R≤r. 

The major processing steps are as follows:

1. $R_{s}$ is generated by the natural cubic splines using Equations (12) and (13).
2. $R_{s}$ and the initial value of $\alpha_{S}$ are added into Equation (17) to calculate $\alpha_{S}$.
3. $R_{s}$ and $\alpha_{S}$ are added into Equation(16) to estimate the systematic error $\hat{s}$.
4. Airborne gravity disturbances subtract $\hat{s}$ to get the airborne gravity disturbances without the systematic errors.
5. The airborne gravity disturbances without the systematic errors are brought into Equation (19) to calculate $\alpha_{R}$.
6. Finally, the ground gravity disturbances are obtained by Equation (21).

## 3. The Louisiana Project: The Experimental Results 

For the purpose of demonstrating that the semi-parametric method can estimate the systematic observation errors without the external surface gravity data, we show four cases:

Case a: The inverse Poisson’s integral
Case b: The semi-parametric method
Case c: The regularization
Case d: The semi-parametric method combined with regularization

In all these experiments we will downward continue the gravity disturbances from approximately 11 km to 0 km (sea level). All these downward continuation cases are based on the remove-compute-restore approach with 360° of the gravity field from EGM2008 [37].

### 3.1. The Data Description

In 2008 the NGS of the USA National Oceanic and Atmospheric Administration (NOAA) launched the GRAV-D project for the airborne gravity surveys for the geoid determination. The data from the Louisiana block survey was released in 2013. The block is 430 km by 460 km, located in the Gulf of Mexico, covering the coastal areas of Louisiana and the ocean areas from 200 to 300 km offshore. The area is defined by the latitude between 27° and 31° and the longitude between 269° and 273.5° [7,14].

**Figure 1:**
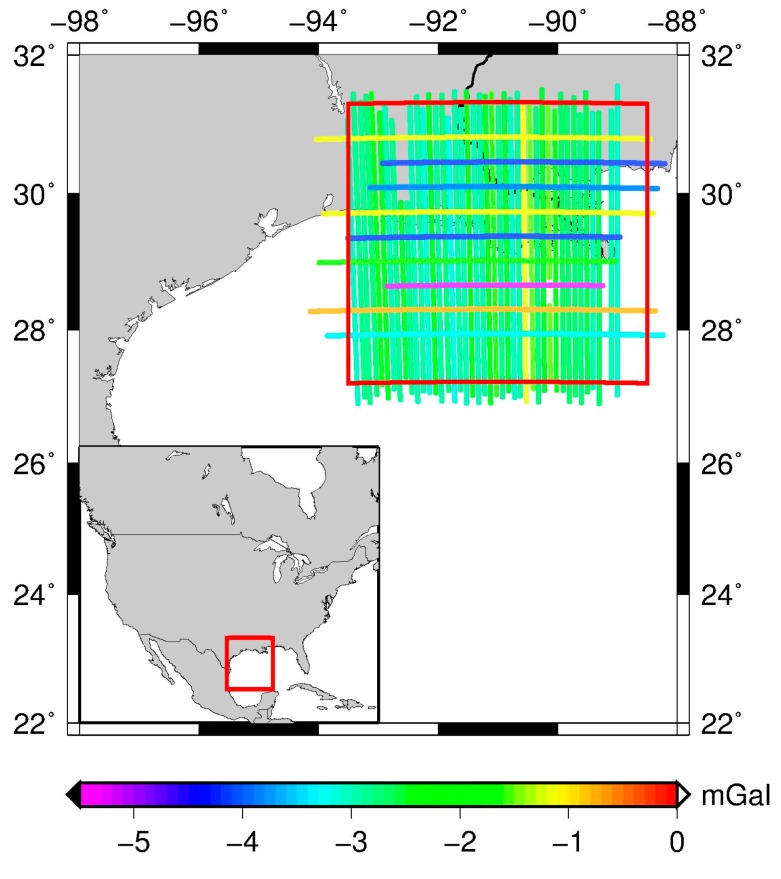
The distribution of the bias from EGM08 of the airborne gravimetry lines in Louisiana (the North-South lines are the survey lines, the West-East lines are the cross lines with the significant errors).

There was an apparent bias (Figure 1 and Table 1) in the Louisiana survey results, so an additional correction was applied before the crossover analysis to adjust the mean gravity value of each line to the mean gravity value of EGM08 (up to 2190°) along this line. The way to estimate the absolute bias of the Louisiana data can use the independent satellite global gravity models (GGMs). The mean values of the difference between EGM08, satellite GGMs and the Louisiana air gravity disturbances are about 2–3 mgal in Table 2. The DIR-R5 [38] model and TIM-R5 [39] model used are 220°. And GOCO05C [40] model used is 720°. Table 1 shows a bias of 2–3 mgal in the overall data set.

The bias-corrected difference between the cross line gravity values and the main N-S data lines gravity values are the residuals. The root mean square (RMS) of the residuals yields the total RMS error. The result of the crossover analysis is shown in Table 2.

### 3.2. The Test Results and the Analysis

The error in the aerogravimetric surveys can adequately described as a linear combination of a random error, the bias, the linear drift and the periodic error [11]. Therefore the simulated drifts and periodic errors are added to the observations to ensure that all error types are present in the numerical experiment and to illustrate the effectiveness of the semi-parametric method in dealing with all kinds of the systematic errors. We use a drift rate of 0.002 mgal/s. The frequency of the periodic error (a sine function) is 0.003 Hz and the amplitude is 6 mgal. These parameters are quoted in [11] based on the analysis of relationship between the gravity measurement system and the systematic errors.

Next we design the numerical experiment consisting of four tests (see Section 2.4) The gravity disturbances at sea level are calculated from EGM08 as the control data (the “original values”) keeping in mind the high accuracy of EGM08 in North America.

**Figure 2:**
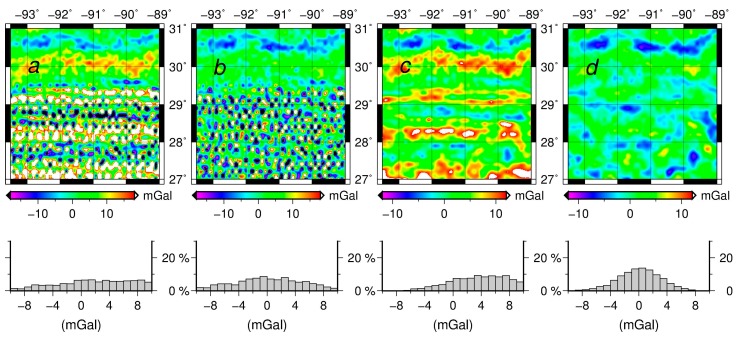
The distribution and the histograms of the differences between the downward continued values and the original values in the Louisiana Gravity Project (**a**) The inverse Poisson’s integral; (**b**) The semi-parametric method; (**c**) The regularization; (**d**) The semi-parametric method combined with the regularization.

**Figure 3:**
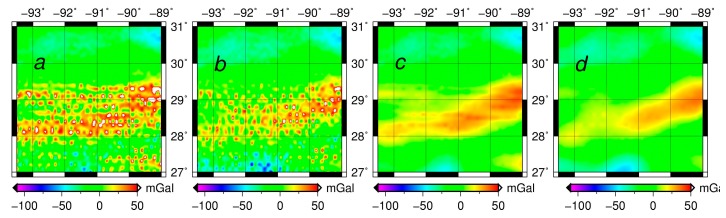
The distribution of the downward continued values in Louisiana Gravity Project (**a**) The inverse Poisson’s integral; (**b**) The semi-parametric method; (**c**) The regularization; (**d**) The semi-parametric method combined with regularization.

The inverse Poisson’s integral, was found to yield the downward continued gravity disturbances containing large random and systematic errors in Table 3 and Figure 2. The results (Figure 3) of purely semi-parametric method seem to have less systematic errors, but the effect of random noise are large (see Figure 2b). It is concluded that the semi-parametric model without the external high-accuracy gravity data works effectively to reduce the systematic errors. 

For the regularization method (see Figure 2c,d), the random errors are reduced significantly. However the semi-parametric method combined with the regularization is more efficient than the regularization method alone and the semi-parametric method alone respectively. The results illustrate that the regularization method can reduce most of the random errors as seen in Figure 2a–c and in Figure 2b–d. We also see that the semi-parametric method eliminates the systematic errors effectively, as seen in Figure 2a,b and in Figure 2c,d. The smoothing parameter is 1.007 and the regularization parameter is 0.504.

## 4. The Tibetan Plateau Experimental Results 

### 4.1. Data Description

**Figure 4:**
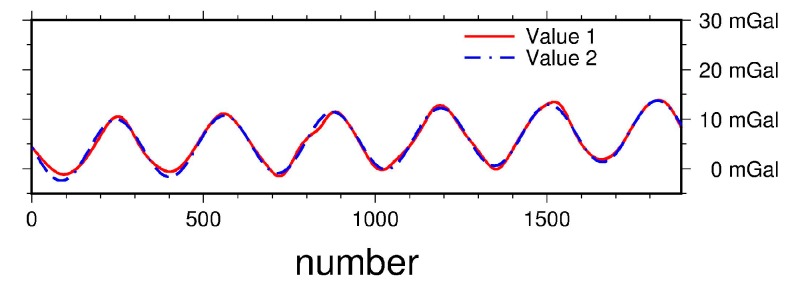
The systematic errors in the Tibetan Plateau (Value 1: The simulated systematic error; Value 2: The estimated systematic error from the semi-parametric model).

As for the Tibetan Plateau, where the surface gravity data are not available, the airborne gravity disturbances and the ground gravity disturbances are obtained using EGM2008. This is just to have a rough approximation of the true field which, nevertheless, is consistent both at the topography and at the flight height. EGM2008 is of course not a perfect approximation of the true field for the Tibetan Plateau. However, the purpose of this numerical experiment is the noise suppression in downward continuation for the extreme but known topography of the Tibetan Plateau. If the EGM2008 model will be included in the future Tibetan Plateau aerogravity project, the errors of EGM2008 should also be included. The simulated error data consist of the random errors and the systematic errors. The standard deviation of the random errors is 2 mgal and the simulated systematic errors contain the bias (3 mgal), the drift and the periodic errors. The rate of drift is 0.002 mgal/s. The frequency of the periodic error is 0.003 Hz, and the amplitude is 6 mgal. The sine function is used to represent the periodic function. The statistics are shown in Table 4 and Figure 4.

### 4.2. Test Results and the Analysis

**Figure 5:**
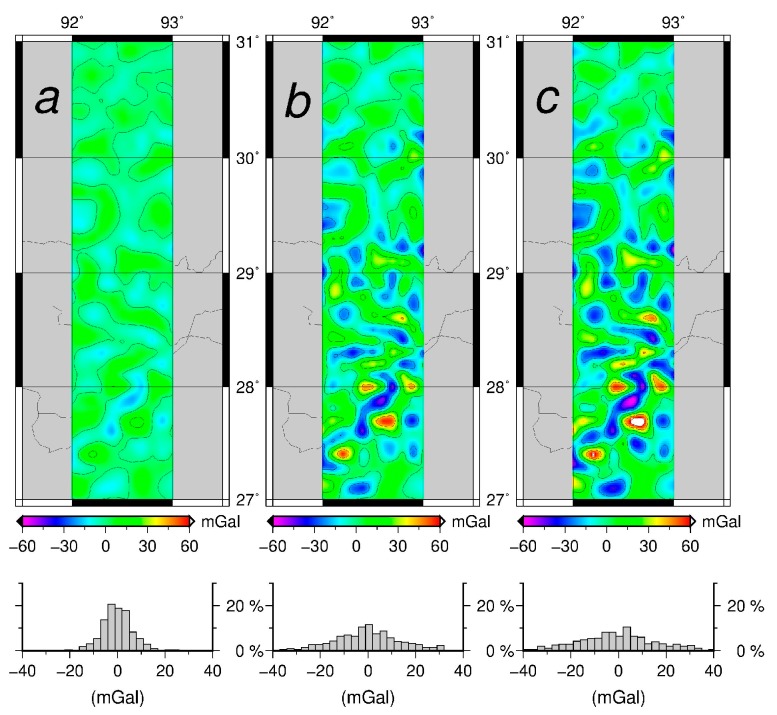
Distributions and histograms of differences between the downward continued values and the original values in the Tibetan Plateau (**a**) 11.5 km to 5.5 km; (**b**) 5.5 km to 0 km; (**c**) 11.5 to 0 km.

**Figure 6:**
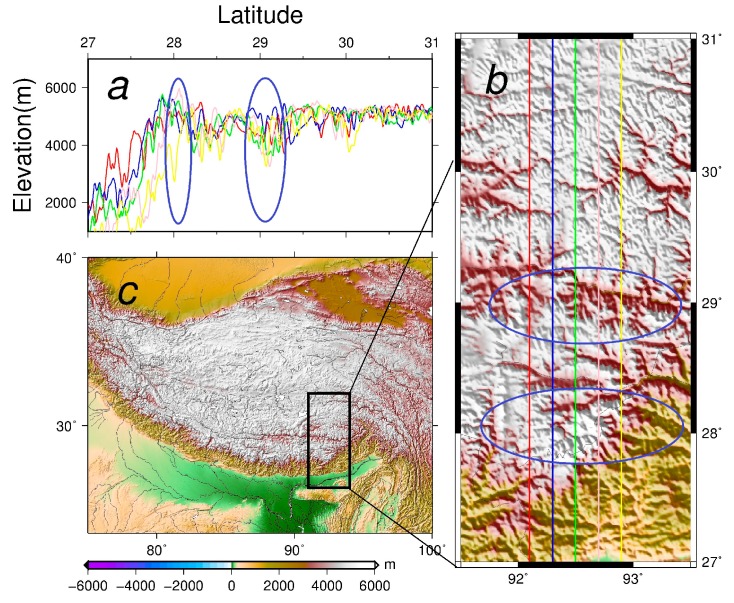
The topography profiles and the roughness of the topography in the Tibetan Plateau (**a**) profile altitudes at different longitudes, the blue ellipse in Figure 6a and Figure 6b the areas analyzed; (**b**) the topography of the computation area, the different color straight lines are located in different longitudes as well as Figure 6a; (**c**) the overview of the topography of the Tibetan Plateau, the black rectangle is the boundary of the computation area.

The semi-parametric method combined with the regularization generates again a better downward continuation results than any other method (Table 5). The inverse Poisson’s integral method, as expected, could not remove the effect of errors, and the RMS of the differences between the downward continued values and the original values is the biggest. The regularization could reduce the random error effect effectively but the bias couldn’t be eliminated. This can be seen from the mean values of the differences. The semi-parametric method combined with the regularization proposed in this paper could suppress both the systematic errors and random errors - as seen from the RMS of differences between the downward continued values and the original values (Figure 5c). In other words, the maximal value of the difference is reduced significantly when comparing the statistics of the inverse Poisson’s integral and the semi-parametric method combined with the regularization. To make sure that the proposed method can estimate the systematic errors effectively, we compare the estimated values and the original EGM08 values (Figure 6). The statistics of the simulated systematic errors and the estimated values are very close. The RMS of the difference is about 0.5 mgal which is much smaller than the current airborne gravimetry error level. The smoothing parameter is 0.020 and regularization parameter is 2.529.

### 4.3. Test and Analysis of the Influence of the Flight Altitude

Compared to the Louisiana project in Section 3, the RMS of the differences in the Tibetan Plateau area are larger. The maximum difference of the new method in Tibet is 70.939 mgal, compared to 9.689 mgal in Louisiana. The errors of downward continuation results in the Tibetan Plateau are significantly larger than in Louisiana. The reason is obviously connected with the large topographic effect in Tibet, see [7]. To confirm that the downward continuation errors of our experiment for the Tibetan Plateau experiment is caused by the severe terrain roughness, we show the topography profiles in this area (Figure 6). The areas where the topography is rough co-locate with the areas where the errors of the semi-parametric method combined with the regularization are the largest (Figure 5), see the area marked by the blue ellipse on Figure 6. 

We conclude that the downward continued errors are mainly caused by the roughness of the topography. It is further confirmed by observing that the errors in the northern part of the area where the topography is smooth are not significant. To further study the flight altitude effect on downward continuation we also make a test with the downward continuation altitude to half the altitude (11.5 km to 5.5 km and 5.5 km to 0 km). The 11.5 km to 5.5 km results (Table 6) show much less errors than the 5.5 km to 0 km results (Table 7). The reason is most probably that the topographical heights in Tibet are about 5000 m. Therefore, the topography effect on the downward continuation in 11.5 km to 5.5 km almost disappears, and the systematic error effect and the random error effects in the two cases are also smaller than the 11.5 to 0 km. In conclusion, the lower downward continuation flight altitude is more suitable in the Tibetan Plateau in order to reduce the error caused by the topography.

## 5. Analysis of the Relationship between the Downward Continuation Errors and the Flight Altitudes of the Tibetan Plateau

The flight altitudes change in different airborne survey projects. In the Tibetan Plateau, the high flight altitude is unavoidable. Therefore, the relationship between the error effect on the downward continuation result and the flight altitude is discussed here. In general, the theoretical derivations of the relationships depend on the error propagation theory.

The basic formula of mixed (systematic and random errors) is:

(22) $$\Omega =\Delta +s=\tilde{L}-L$$

Δ is the random error, s is the systematic error and L is the observation vector. $\tilde{L}$ is the estimated observation vector. The sum of the random errors and the systematic errors is the difference between the original values and the observed values. The comprehensive variance $D_{LL}$ of the observed values mixed systematic errors and random errors is calculated as follows:

(23) $$D_{LL}=MSE(L)=E{\left(L-\tilde{L}\right)}^{2}=E(\Omega )=\sigma^{2}+s^{2}$$

The estimated value mean square error (*MSE*) formulas are derived according to the law of the error propagation assuming that the random and systematic errors are uncorrelated:

(24) $${\hat{X}}_{IPI}={\left(B^{T}PB\right)}^{-1}B^{T}P\delta g^{air}$$

set $J={\left(B^{T}PB\right)}^{-1}B^{T}P$. 

*MSE* of the estimated value is calculated by the following equation:

(25) $$MSE(\hat{X})=E({\left(\hat{X}-X\right)}^{T}(\hat{X}-X))$$

It can be expressed as follows:

(26) $$MSE(\hat{X})=tr(D(\hat{X}))+tr{\left[(E(\hat{X})-X)(E(\hat{X})-X)\right]}^{T}$$

Then the inverse Poisson’s integral $MSE({\hat{X}}_{IPI})$ yields:

(27) $$MSE({\hat{X}}_{IPI})=\sigma_{0}^{2}tr(JQJ^{T})+tr(Jss^{T}J^{T})$$

Therefore, it is clear that $MSE({\hat{X}}_{IPI})$ consist of two terms according to the Equation (27). For $\sigma_{0}^{2}tr(JQJ^{T})$, it is the random error contribution to the *MSE* while $tr(Jss^{T}J)$ is the systematic error contribution to the *MSE*. Since the *RMS* is generated from *MSE*. Thus, *RMS* of the difference between the downward continued values and the original values can be divided into random error effect (*REE*) and the systematic error effect (*SEE*):

(28) $$REE=\sqrt{\sigma_{0}^{2}tr(JQJ^{T})/t}$$

(29) $$SEE=\sqrt{tr(Jss^{T}J^{T})/t}$$

where t is the number of the estimated parameters.

**Figure 7:**
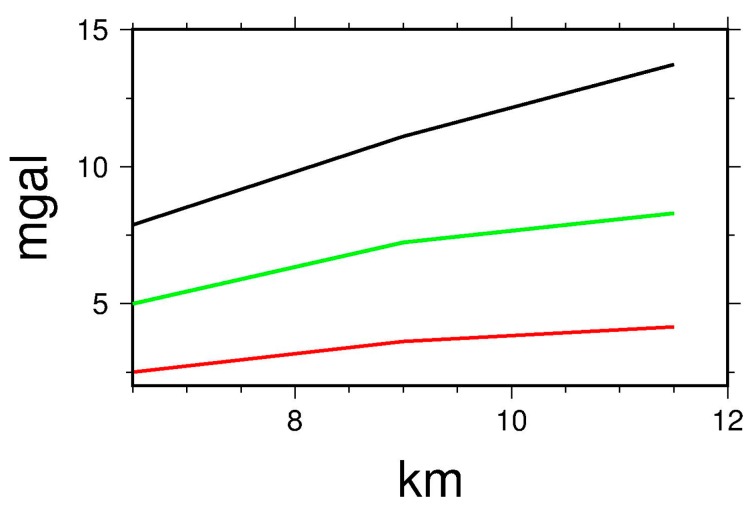
The Relationship between Errors Effect and Downward Continuation Altitude in Tibetan Plateau (Red line: *REE*-Std 2 mgal, green line: *REE*-Std 4 mgal, black line: *SEE*).

In order to analyze *REE* and *SEE* quantitatively some tests with simulated error values are carried out. The standard deviation of the random error in the test is 2 mgal or 4 mgal. And the statistics of the systematic error in the test are as the same as the original values shown in Table 5 and Figure 7. 

The results are shown in Table 8. The *REE* and *SEE* increase as the flight altitude increase. The effect for the flight altitude of 11,500 m is nearly twice as big as the effect for the flight altitude of 6500 m. In conclusion, *REE* and *SEE* are both increasing for increasing flight altitude. And *REE* does not change at the same rate as *SEE*. Therefore, it is necessary to deal with *REE* and *SEE* separately.

## 6. Summary and Concluding Remarks

In this paper we have analyzed different methods for suppressing the systematic errors and the random errors in downward continuation. The numerical tests were done for two areas, Louisiana, (USA) and the Tibetan Plateau (China). In the analysis we have assumed the simulated airborne survey errors on both real survey data (Louisiana) and the simulated survey data (Tibet).

1. We have used four different methods for solving the inverse Poisson’s integral, and found that the semi-parametric method combined with the regularization is the best. The RMS of the difference between the signals downward continued from the flight altitude compared to the ground original EGM08 values is smallest.
2. The airborne gravity data in Louisiana and the simulated data for the Tibetan Plateau both prove that the proposed method works effectively. In addition, the proposed method is not only best for the downward continuation of the measured aero gravimetric data, but also could improve the airborne gravity data accuracy for the parts of the airborne surveys which are poorly determined.

In the future, we can expect improvements in the airborne gravimetry using the Strapdown Inertial Navigation System and the Unmanned Aerial Vehicle airborne gravity surveys at different altitudes, although such systems will often have systematic errors and random errors, e.g., due to accelerometer and gyro drifts. More detailed future studies should include test the areas which are mountainous and where the surface gravity is known. The proposed concept of downward continuation could be very useful for such applications.

## Figures and Tables

**Table 1:** Difference between GGMs derived values and the airborne gravity disturbances of the Louisiana gravity project (mgal).

	EGM08		DIR-R5		TIM-R5		GOCO 05C	
	Mean	Std	Mean	Std	Mean	Std	Mean	Std
North-South lines	−2.474	0.445	−2.339	4.940	−2.381	4.872	−2.434	1.651
West-East lines	−2.873	1.668	−3.018	5.486	−3.077	5.459	−2.893	2.258

**Table 2:** Statistics of crossover error analysis in Louisiana.

Items	Values
Altitude (m)	11 088
Number of Crossovers	361
RMS of Residuls (mgal)	1.8
Std of Resduals (mgal)	1.8
Mean Crossover Difference (mgal)	−0.19
RMS Error (mgal)	1.28

**Table 3:** Statistics of the differences between the downward continued values and the original values in the Louisiana Gravity Project (mgal).

	Max	Min	Mean	RMS
The inverse Poisson’s integral	117.154	−57.081	6.858	19.331
The semi-parametric model	64.589	−61.587	0.141	14.167
The regularization	18.543	−8.554	4.255	6.060
The semi-parametric method combined with the regularization	9.689	−9.722	0.110	2.922

**Table 4:** The statistics of the systematic errors in the Tibetan Plateau (mgal).

	Max	Min	Mean	Std
Simulated values	13.713	−2.436	5.621	4.598
Estimated values	13.789	−1.548	5.751	4.478
Differences	1.612	−1.506	−0.129	0.558

**Table 5:** Statistics of the differences between the downward continued values and the original EGM08 values in the Tibetan Plateau (mgal).

	Max	Min	Mean	RMS
The inverse Poisson’s integral	143.684	−53.674	6.212	22.908
The semi-parametric model	70.838	−56.561	−0.440	19.223
The regularization	73.519	−53.697	1.686	20.002
The semi-parametric method combined with the regularization	70.939	−56.596	−0.429	19.139

**Table 6:** The statistics of the differences between the downward continued values (from 11.5 km to 5.5 km) and the original values in the Tibetan Plateau (mgal).

	Max	Min	Mean	RMS
The inverse Poisson’s integral	27.832	−19.121	4.364	8.366
The semi-parametric model combined with the regularization	24.913	−19.865	−0.022	6.428

**Table 7:** The statistics of the differences between the downward continued values (from 5.5 km to 0 km) and the original values in the Tibetan Plateau (mgal).

	Max	Min	Mean	RMS
The inverse Poisson’s integral	59.829	−44.107	4.466	16.673
The semi-parametric model combined with the regularization	56.830	−47.562	−0.157	15.669

**Table 8:** Errors Effect and downward continuation altitude in Tibetan Plateau (mgal).

Altitude (m)	*REE*		*SEE*
	2 mgal (Std)	4 mgal (Std)	
6500	2.495	4.991	7.873
9000	3.618	7.236	11.108
11,500	4.013	8.027	13.726
